# SGLT2 Inhibition Protects Cognitive Function in Alzheimer's Disease by Enhancing Brain Perfusion, Independent of Glucose Control

**DOI:** 10.1002/alz70855_096815

**Published:** 2025-12-23

**Authors:** Andrew P. Gregory, Gilbert Charles Morgan, Chengyun Tang, Huawei Zhang, Jane J. Border, Xing Fang, Richard J. Roman, Fan Fan

**Affiliations:** ^1^ Augusta University, Augusta, GA, USA; ^2^ University of Mississippi Medical Center, Jackson, MS, USA

## Abstract

**Background:**

SGLT2 inhibitors were initially approved by the FDA to lower blood sugar in type 2 diabetes. Later clinical trials showed significant benefits in treating heart failure, even in patients without uncontrolled diabetes. Recent evidence highlights cerebral hypoperfusion as an early symptom of AD/ADRD. We have previously shown that SGLT2 inhibitors improve brain perfusion and cognition in diabetes‐related ADRD rats. This study aims to explore whether SGLT2 inhibition also benefits brain perfusion and cognition in AD, independent of glucose control.

**Methods:**

To investigate the effects of SGLT2 inhibition, TgF344‐AD rats were administered luseogliflozin orally (20 mg/kg/day) for 3 months. Cognitive function was evaluated using an eight‐arm water maze, while cerebral vascular function was assessed through pressure myography to measure the myogenic response of the middle cerebral artery and laser‐Doppler flowmetry to evaluate cerebral blood flow autoregulation. Additionally, the transcriptomic profile was obtained from bulk RNA‐seq analysis conducted on primary VSMCs isolated from AD rats treated with either luseogliflozin or vehicle.

**Results:**

Body weights, plasma glucose, and HbA1c levels were unaltered in vehicle and drug‐treated AD rats. SGLT2 inhibition improved learning and memory in AD rats. Impaired myogenic responses and cerebral blood flow autoregulation observed in AD rats were normalized with SGLT2 inhibition, associated with restored molecular pathways essential for VSMC contractility and vascular function.

**Conclusions:**

These results indicate that SGLT2 inhibition protects cognitive function in AD by enhancing cerebral blood flow autoregulation, highlighting a mechanism that involves improved cerebrovascular function rather than glucose regulation. Our study opens new avenues for targeting cerebral perfusion as a treatment strategy for AD/ADRD.

**Financial Disclosure**: None.

**Funding Resources**: This study was supported by grants AG079336, AG057842, P20GM104357, and HL138685 from the National Institutes of Health, TRIBA Faculty Startup Fund from Augusta University, and 25PRE1365157 from the American Heart Association.

